# Spontaneous blood pressure reduction of previously hypertensive patients as a symptom of central pulmonary artery embolism

**DOI:** 10.1007/s00392-023-02315-z

**Published:** 2023-10-05

**Authors:** R. Schell, F. Alban, N. Frey, C. Erbel

**Affiliations:** 1 Department of Internal Medicine III, Division of Cardiology, University Hospital Heidelberg, Ruprecht-Karl University Heidelberg, Im Neuenheimer Feld 410, 69120 Heidelberg, Germany; 2 DZHK (German Center for Cardiovascular Research), Partner Site Heidelberg/Mannheim, Heidelberg, Germany

Sirs,

Venous thromboembolism presents as pulmonary artery embolism and/or thrombosis of the deep (leg) veins and represents together with acute coronary syndrome and stroke one of the three most common acute cardiovascular events [1]. The incidences for pulmonary artery embolism published in the epidemiologic literature range from 39 to 115 per 100,000 population [1, 2]. The wide range of these values may be explained by one of the central and most remarkable features of pulmonary artery embolism; venous thromboembolism often is asymptomatic or nearly so, subject to misdiagnosis, and thus remains undetected even in fatal cases [3]. The resulting limitations in terms of incidence and its evolution over time are obvious, but more serious is the associated challenge of timely diagnosis, risk stratification, and appropriate treatment initiation.

We here report a series of three patients who were treated with central pulmonary artery embolism in our department during the past months. All three patients presented with a blood pressure reduction of preexisting arterial hypertension as a remarkably common symptom leading to complete discontinuation of oral antihypertensive medication in the days or up to 3 weeks prior to hospital admission. As a representative example of this observation, the case report of one of these patients will be described here in detail.

A 66-year-old female patient with preexisting arterial hypertension presented to the emergency department after referral by her primary care physician for new onset of shortness of breath. The patient reported that she had undergone elective outpatient surgery for claw toe 8 weeks before the time of admission and had been immobilized as a result. This immobilization had been accompanied by thromboprophylactic therapy with low-molecular-weight heparin. In the last 4 days before presentation in the emergency room, there had been progressive shortness of breath upon the lightest exertion as well as thoracic complaints. In retrospect, it was noticeable that since about 3 weeks before, blood pressure self-measurements with pre-existing arterial hypertension under four-fold therapy yielded hypotensive blood pressure values with systolic values around 100 mmHg. Hypotension had been symptomatic only on a few days with dizziness and subjectively reduced performance, and had led the general practitioner to gradually reduce and ultimately completely discontinue the longstanding antihypertensive medication. The medication had previously been taken for years, consisting of an Angiotensin II receptor blocker (ARB), a beta blocker, a loop diuretic and hydrochlorothiazide (HCTZ). There were no other symptoms, in particular tachycardia, palpitations, or syncope/collapse. Comorbidities were hypercholesterolemia and former nicotine abuse with 30 packyears, an unspecified obstructive airway disease treated with a combination of inhaled steroids and a beta agonist, as well as grade I obesity (body mass index 29.8 kg/m^2^).

**Fig. 1:**
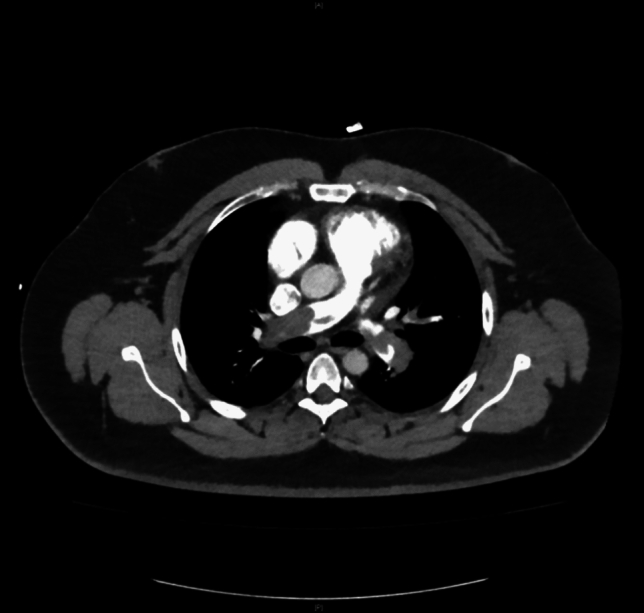
CTPA of 66 year old female patient with bilateral pulmonary artery embolism with subtotal occlusion of both pulmonary arteries

**Fig. 2:**
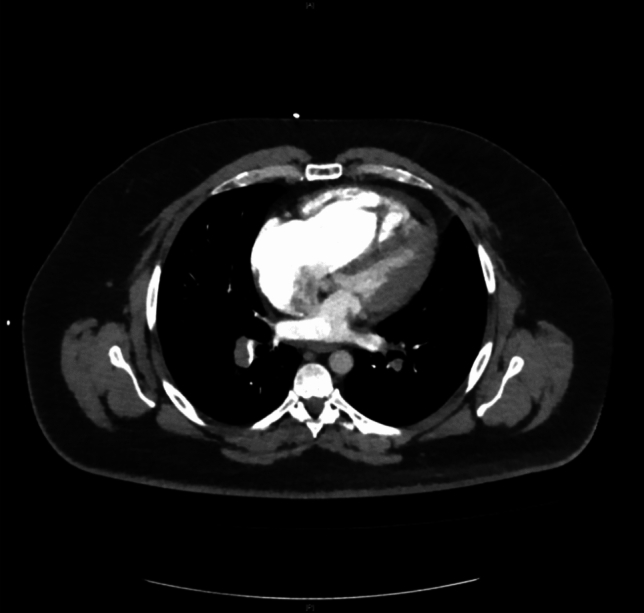
Same patient as Fig. 1, CTPA demonstrating signs of increased right ventricular strain with RV/LV ratio significantly >1

**Fig. 3:**
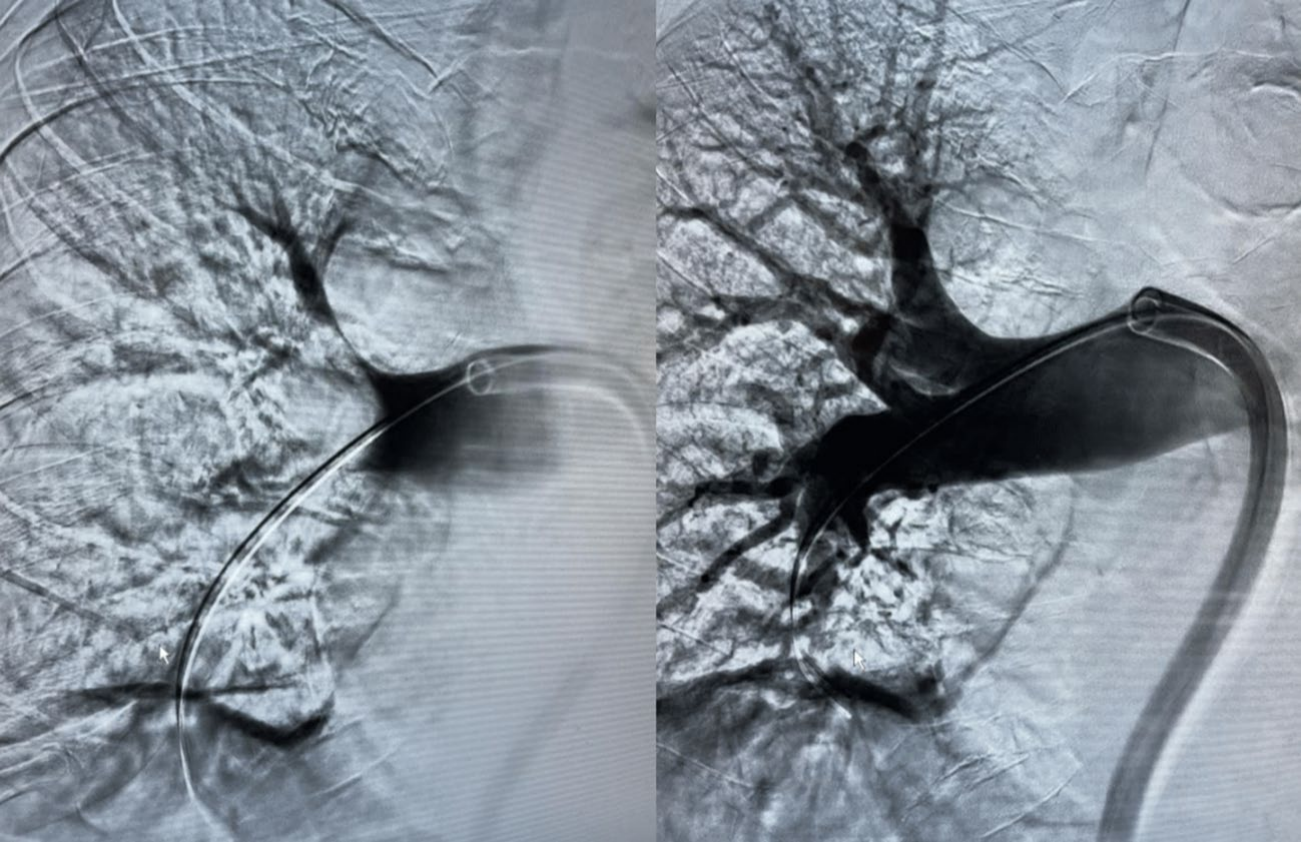
DSA demonstrating a large central thrombus in the right pulmonary artery before (left side) and after (right side) thrombectomy

**Fig. 4:**
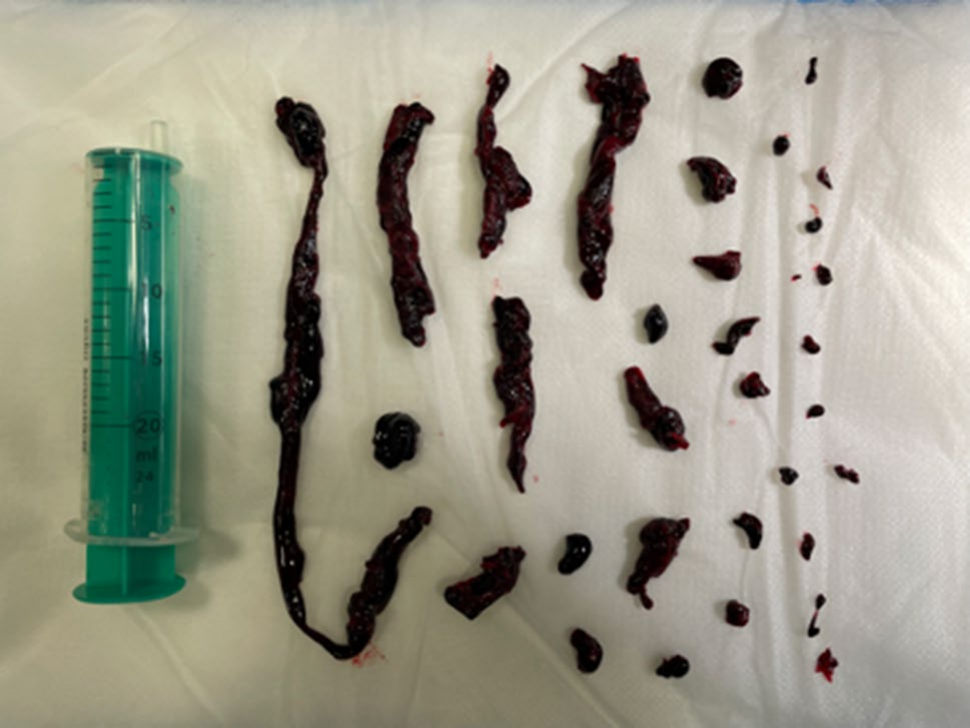
Multiple removed thrombi and fragments

Presentation to the emergency department revealed a hemodynamically stable patient with borderline tachycardia (heart rate 102/min), an inconspicuous blood pressure of 120/70 mmHg and resting dyspnea (breathing rate 18/min) with pulse oxymetric oxygen saturation of 94% under 4 l of oxygen by nasal cannula. Physical examination revealed no significant abnormalities apart from slightly prolonged expiratory flow with bilateral vesicular breath sounds and a discrete swelling of the right leg. Electrocardiography showed no abnormalities apart from the discrete sinus tachycardia described above. Laboratory chemistry revealed an elevation of hsTroponinT (94.1 pg/ml; norm value < 14 pg/ml) as well as D-dimer (11.5 mg/l; norm value < 0.5 mg/l) and NTproBNP (623 ng/l; norm value < 450 ng/l); furthermore, a discrete inflammatory constellation (CRP 20.3 mg/l; norm value < 2 mg/l, leukocytes 10.49/nl; norm values 4–10/nl) was seen with otherwise essentially regular results. Transthoracic echocardiography (TTE) revealed dilatation of the right ventricle (RV) with reduced RV function and a noninvasively estimated systolic pulmonary artery (PA) pressure of 55 mmHg with good left ventricular pump function (EF 58%). With history, clinical, laboratory, and echocardiographic suspicion of the presence of pulmonary artery embolism, computed tomography (CT) angiography was promptly performed. CTPA (computed tomography pulmonary angiography) revealed bilateral pulmonary artery embolism with subtotal occlusion especially of the right pulmonary artery, and complete to incomplete occlusion of single lobe and segmental arteries distal thereto in the upper, middle, and lower lobes, as well as signs of increased right ventricular strain with an RV/LV ratio significantly > 1 (Figs. 1 and 2). The pulmonary embolism response team (PERT) was involved and, after informed consent, interventional thrombus aspiration was performed with the FlowTriever device (Inari Medical, California) in bilateral central pulmonary artery embolism of the intermediate high-risk category (simplified pulmonary embolism severity index (sPESI) = 1, positive biomarkers, right heart strain signs on CT and TTE). Invasive measurement preprocedurally confirmed an increase in PA (pulmonary arterial) pressure (systolic/diastolic/mean = 56/24/32 mmHg) and a large thrombus in the proximal right pulmonary artery (Fig. 3). After four aspirations on the right and two aspirations on the left side with the 24F Triever Aspiration Catheter, a number of thrombus fragments with subacute and older or already connective tissue remodeled and fresh portions (Fig. 4) could be aspirated. Postprocedurally, there was an immediate decrease in pulmonary arterial pressure to systolic/diastolic/mean = 44/20/28 mmHg and remarkable clinical improvement. Respiratory rate normalized, oxygen supplementation could be discontinued, pulse oximetry measured oxygen saturation of 98% under room air, and heart rate reduced to 74/min already periprocedurally. In the next days, final clinical workup, no intraluminal thrombotic material of the deep pelvic and leg vein system could be detected; echocardiography also showed normalization of RV diameter and function and estimated PA pressure. Vital signs at discharge were normal (blood pressure 118/68 mmHg, heart rate 86/min, SaO_2_ 99%). The patient was started on a new oral anticoagulant after pretreatment with anti-Xa-controlled dosing of low-molecular-weight heparin and could be discharged to further outpatient care after 2 days. Here, the previously discontinued antihypertensive medication was gradually reimplemented, under which well-controlled blood pressure values could be documented.

Another patient of the described case series (75 years old, male, status post pulmonary artery embolism in 2005) had completely discontinued his antihypertensive medication (combination preparation of ARB, calcium antagonist and HCTZ) 4 days prior to presenting to the emergency department. He complained about fatigue and had documented blood pressure values < 120 mmHg systolic twice. Diagnosis revealed bilateral central pulmonary artery embolism with elevated cardiac biomarkers and echocardiographic signs of right heart strain (sPESI = 0), which already diminished after mechanical thrombectomy during hospitalization. The third case in the series described was a 69-year-old female patient with known chronic obstructive pulmonary disease and home oxygen therapy who complained of a nonspecific worsening of her condition continuously for 2 weeks and subsequently had her antihypertensive medication (ACE-inhibitor (angiotensin converting enzyme inhibitor) and calcium antagonist) halved in dose in response to multiple hypotensive blood pressure readings taken by her primary care physician. Presentation by the ambulance service revealed a pulse oxymetric oxygen saturation of 82% and uncompensated respiratory alkalosis on the basis of unilateral pulmonary artery embolism with subtotal occlusion of the right pulmonary artery (sPESI = 2). The thrombus tissue removed during subsequent interventional thrombectomy showed partially older and incipient connective tissue remodeled portions; oxygen demand reduced postinterventionally back to the preexisting flow rate.

The described cases demonstrate an exemplary and noteworthy procedure of a diagnostic algorithm in case of already anamnestic suspicion of pulmonary artery embolism as well as a guideline-based risk stratification with individualized decision for interventional therapy. The interventional procedure in terms of mechanical thrombectomy could safely and remarkably effective achieve an immediate clinical benefit as well as improvement of laboratory and imaging parameters. At the same time, the case is based on a subacute course with presumably several pulmonary embolic events, each of which was already discretely or rather atypically symptomatic. In retrospect, the blood pressure reduction of previously hypertensive patients progressing to symptomatic hypotension, which resulted in the pause and discontinuation of the previously regularly taken antihypertensive medication, needs to be interpreted as an (early) symptom of pulmonary artery embolism. Pathophysiologically, the mechanism can be interpreted as a moderate or subacute form of left heart forward failure: as a result of partial or complete obstruction of the lumen of one or more pulmonary arteries, there is increased RV afterload, which, in addition to RV dilatation and increased wall stress, neurohumoral activation, (right ventricular) ischemia, and resultant RV dysfunction and failure, sequentially or simultaneously, leads to a reduction in left ventricular preload and consequently cardiac output.

The details of the case described and of the case series not detailed here for reasons of scope and redundancy, which showed striking similarities to the patient presented, underscore the symptomatic diversity of pulmonary artery embolism. On one hand, the wide spectrum of possible—and of course often in free combination occurring—symptoms of shortness of breath, chest pain or thoracic tightness, tachycardia, hypotension, collapse or syncope, dizziness, tachypnea and/or cyanosis, cough, hemoptysis, and (neck) venous congestion; on the other hand, there are symptoms of variable severity, often with an oligosymptomatic course or with a completely asymptomatic clinical picture. For example, a structured diagnostic workup of patients hospitalized with first-time syncope demonstrated pulmonary artery emboli with a prevalence of approximately one in six patients [4]. From this and in analogy to the case series described here, it can be concluded that even supposedly atypical signs, such as the described blood pressure reduction of previously hypertensive and drug-treated patients, should definitely be appreciated and evaluated as a potential symptom. This is especially true in situations of special risk constellations such as the immobilization described here. In retrospective analysis, thromboprophylaxis with low-molecular-weight heparin proved ineffective and should not lead to early exclusion of thromboembolism in differential diagnoses. The incidence of venous thromboembolic events under existing thromboprophylaxis cannot be neglected, as recent data from the COVID-19 pandemic in particular emphasize [5], and plays an even more relevant role in critically ill patients [6, 7]. The presence of pulmonary artery embolism should also be suspected when initiated therapy of another (suspected) disease, especially of the respiratory system, does not lead to clinical improvement.

According to a recently published interim analysis of the FLASH registry (FlowTriever All-Comer Registry for Patient Safety and Hemodynamics), approximately one third of intermediate-high-risk PE patients present in a hemodynamically stable state at first sight in terms of regular blood pressure values, but show a reduced cardiac index (CI) [8]. This group has been described as normotensive shock and is suspected to be at particular risk of hemodynamic decompensation and consequently higher mortality. Nevertheless, this subgroup can only be unambiguously identified by performing a right heart catheterization to determine the CI, which is why a composite shock score was determined to stratify patients by clinically easily determined criteria. In this evaluation, analysis showed that when all criteria of the composite shock score (increased TNT, increased BNP, reduced RV function, saddle PE, concomitant DVT, and tachycardia) were met, there was a significant prediction and prevalence of normotensive shock of nearly 60%. Despite these important findings, albeit only retrospectively collected, no data are yet available to answer the question of whether patients with normotensive shock differ in outcome compared with patients without normotensive shock. Pathophysiologically unconvincing is the aspect that the patients with normotensive shock did not have higher lactate levels. The mechanism of increased RV afterload due to pulmonary obstruction leading to RV dilatation and increased wall tension, neurohumoral activation, (right ventricular) ischemia, and resulting RV dysfunction and failure explains shock well. However, as a result of tissue hypoxia, this would lead to predominantly anaerobic metabolism and accumulation of lactate, the importance of which to prognosis and predictive probability for PE-associated complications in the acute phase of pulmonary artery embolism has been demonstrated many times. Ultimately, despite the statistically significant predictive value of the composite shock score, the question remains whether a prevalence of less than 60% despite the presence of all criteria is sufficient to form a clinically useful score and to gain importance in clinical practice. Despite all limitations and criticisms, further studies will undoubtedly be needed to determine (i) whether more aggressive management with thrombolytic therapy and/or mechanical thrombectomy can improve the outcome of patients with intermediate-risk PE and normotensive shock and (ii) whether there are even better clinical parameters to be evaluated, that can predict the presence of normotensive shock or, alternatively, that all patients with intermediate-high-risk PE and a composite shock score > 0 should undergo right heart catheterization testing, is urgently needed.

In summary, pulmonary artery embolism, with pronounced symptomatic variance, potentially serious consequences in terms of morbidity and mortality, and ultimately the aspect that a definitive diagnosis or exclusion can only be made with sufficient certainty by means of CT pulmonary angiography, still represents a particular challenge in everyday clinical practice. Fortunately, CTPA is a diagnostic method that is now widely available, but it should not be used in an unselected manner, both in terms of patient-related factors to avoid undesirable side effects such as the required radiation dose and the use of contrast media, as well as for economic reasons. Meta-analyses show that in emergency departments in the United States, pulmonary artery embolism can be detected in only 1 of 20 patients undergoing CT pulmonary angiography with this question [9]. Before using CTPA examinations, it should be considered that noninvasive tests, which are based on a determination of clinical pretest probability, can efficiently and safely reduce the rate of necessary cross-sectional imaging examinations [10]. Although CT pulmonary angiography is widely accepted as the gold standard, incidences of false-positive findings, which vary widely between centers, have been reported to be as high as 5% [11, 12].

Pulmonary artery embolism remains challenging to diagnose despite significant advances. Especially in view of reports and studies on supposedly atypical symptoms beyond “classical” complaints such as dyspnea and chest pain, the presence of pulmonary artery embolism should be included in the differential diagnostic considerations, particularly in risk constellations. We postulate that blood pressure reduction in previously hypertensive patients as a symptom of (central) pulmonary artery embolism should be taken into account in differential diagnostic considerations. The workup of patients with suspected or possible presence of pulmonary artery embolism should systematically inquire about antihypertensive drug reduction. The initiation of therapy depending on the appropriate risk stratification aims in particular to prevent recurrent venous thromboembolism as well as hemodynamic and respiratory decompensation [13]. Patients who are categorized as high-risk or intermediate-high-risk in the risk stratification must be evaluated with regard to an indication for systemic fibrinolysis, taking into account the accompanying potentially fatal bleeding risks and contraindications. In particular, for patients who are assigned to the intermediate-high-risk category, as described in the case series, there is no recommendation for the use of a systemic thrombolytic regimen, considering the current data [13–15]. At the same time, data from studies with patients in the intermediate-high-risk category show that in the first seven days after diagnosis, more than 5% of those affected decompensate hemodynamically or even die [14]. In this indication, among others, percutaneous mechanical thrombectomy by means of a large-lumen aspiration catheter seems to be an effective and safe therapy [16–20], which—as in the case described—can achieve an immediate clinical and, with regard to vital signs as well as imaging, demonstrable improvement of the findings even in subacute courses.
